# Analysis of speech and tongue motion in normal and post-glossectomy speaker using cine MRI

**DOI:** 10.1590/1678-775720150421

**Published:** 2016

**Authors:** Jinhee Ha, Iel-yong Sung, Jang-ho Son, Maureen Stone, Robert Ord, Yeong-cheol Cho

**Affiliations:** 1 University of Ulsan, Ulsan University Hospital, College of Medicine, Department of Dentistry, Ulsan, South Korea.; 2 University of Ulsan, Ulsan University Hospital, College of Medicine, Department of Oral and Maxillofacial Surgery, Ulsan, South Korea.; 3 University of Maryland, Department of Oral and Craniofacial Biological Science, Baltimore, United States of America.; 4 University of Maryland, Department of Oral and Maxillofacial Surgery, Baltimore, United States of America.

**Keywords:** Glossectomy, Cine MRI, Tongue, Speech

## Abstract

**Objective:**

Since the tongue is the oral structure responsible for mastication, pronunciation, and swallowing functions, patients who undergo glossectomy can be affected in various aspects of these functions. The vowel /i/ uses the tongue shape, whereas /u/ uses tongue and lip shapes. The purpose of this study is to investigate the morphological changes of the tongue and the adaptation of pronunciation using cine MRI for speech of patients who undergo glossectomy.

**Material and Methods:**

Twenty-three controls (11 males and 12 females) and 13 patients (eight males and five females) volunteered to participate in the experiment. The patients underwent glossectomy surgery for T1 or T2 lateral lingual tumors. The speech tasks “a souk” and “a geese” were spoken by all subjects providing data for the vowels /u/ and /i/. Cine MRI and speech acoustics were recorded and measured to compare the changes in the tongue with vowel acoustics after surgery. 2D measurements were made of the interlip distance, tongue-palate distance, tongue position (anterior-posterior and superior-inferior), tongue height on the left and right sides, and pharynx size. Vowel formants Fl, F2, and F3 were measured.

**Results:**

The patients had significantly lower F2/Fl ratios (F=5.911, p=0.018), and lower F3/F1 ratios that approached significance. This was seen primarily in the /u/ data. Patients had flatter tongue shapes than controls with a greater effect seen in /u/ than /i/.

**Conclusion:**

The patients showed complex adaptation motion in order to preserve the acoustic integrity of the vowels, and the tongue modified cavity size relationships to maintain the value of the formant frequencies.

## INTRODUCTION

In recent years, speech adaptation has been studied in patients who have received glossectomy surgery for oral cancer^15^. Post-glossectomy articulation may be poor because of irregularity of air flow and reduced palatal contact resulting from irregular deformations of the tongue. Patients may also have limited tongue range of motion, deformation ability, and fibrosis, all of which can reduce speech quality. Studies have isolated several major factors that affect speech quality after glossectomy surgery. Larger tumor size has a more negative impact on patient articulation and swallowing function after surgery^14,23^. Tumor location also impacts articulation quality with the anterior tongue having the biggest impact on articulation quality and the tongue base having the biggest impact on swallowing^10,21^. Tumor invasion and radiation treatment also affect post-glossectomy speech. Patients who underwent surgery plus radiation therapy also showed worse function than patients who only underwent surgery^13^.

In order to restore the extensive tissue losses of the oral cavity when mid and large size tumors are removed, reconstruction may be performed using a radial forearm free flap^17^ or an anterolateral thigh flap^16^. There are still controversies in the value of using a free flap for reconstruction^5,6^. Archontaki, et al.^1^ (2010) reported that the use of a free flap was the best way to improve the quality of life of patients after surgery based on an assessment of function in 20 patients who underwent free flap reconstruction. Chen, et al.^7^ (2002), however, reported that patients who underwent hemiglossectomy and partial glossectomy did not need a flap reconstruction in terms of speech. They found that scar tissue beneath the flap interfered with the articulatory movement of the tongue, and that a primary closure made the articulation more accurate after hemiglossectomy and partial glossectomy. However, Sun, et al.^21^ (2007) reported no difference in the speech degradation of patients who were reconstructed with free flap vs an adjacent flap, and Nicolletti, et al.^13^ (2004) found no difference between primary and flap closure. Instead, they found that preservation of the tip was key to retention of speech quality, and that loss of the tip was as disruptive as a hemitongue glossectomy.

The present paper uses F1 and F2 values for vowels, along with tongue motion patterns, to evaluate tongue function in patients who underwent partial lateral glossectomy. Centralization of vowels has been observed in speakers with glossectomy using F1-F2 plots^4,22^, which implies poorer articulation accuracy and a reduction in intelligibility. Distinctiveness among vowels may be more important than global vowel space in determining vowel intelligibility, since significant expansion of vowel space area can be a product of acoustic changes in just one vowel^12^. The vowel /i/ is often considered very difficult for glossectomy speakers to execute because it requires considerable anterior tongue elevation and a forward tongue body^22^. In an examination of /i/, Whitehill and colleagues found no significant differences in the values of F1 between glossectomy patients and controls, but patients had lower F2 values.

Kaji, et al.^9^ (2007) found differences between post-glossectomy gender differences in the formant frequencies of /i/. In females, F2 and F3 values were reduced for patients regarding controls. In males, F1 values were higher in patients than in controls. They hypothesized that men and women process speech differently after a partial glossectomy.

In recent years, improved imaging methodology has allowed the combined study of structure and movement of the tongue. In the 1950s cinefluorography was used to measure tongue movement^2^, and more recently cineradiography and videofluorography have been used. However, there are limitations in clinical use of X-ray because of the risk of radiation exposure^8^. Other alternatives to X-ray include ultrasound, which provides representations of the tongue in motion^18^ and in 3D^19^. The ultrasound wave does not pose any health risks and can identify the morphological changes of the tongue during speech or swallowing. Rastadmehr, et al.^17^ (2008) used ultrasound to examine tongue velocity during the speech of lateral partial glossectomy patients and reported that a compensatory mechanism worked to increase velocity of the residual tongue^14^. Magnetic Resonance Imaging (MRI) has also been used to observe soft tissue clinically. The use of MRI in speech research began with the recording of steady state vowels using static MRI^3^. Static MRI reveals the anatomy of structures in the vocal tract such as the tongue surface and the vocal tract airway. But, static MRI is limited to quantifying and modeling static features, and cannot be used to track tongue motion during speech^20^. The introduction of cine MRI, which produces a time series of MR images, greatly enhanced the *in vivo* visualization of the tongue's motion during speech.

The purpose of this study is to investigate the morphological changes of the tongue and the adaptation of pronunciation using cine MRI for speech of patients who undergo glossectomy.

## MATERIAL AND METHODS

This was a retrospective study, which examined data that had been collected to study speech production in glossectomies. The present study focused on vowels to ascertain whether sounds that appear to sound normal can show compensatory articulatory strategies, which are different from controls. This study used a 2x2 factorial design with repeated measures, in which the two factors were subject group (glossectomies, controls) and vowel (/i/, /u/). The repeated measures were the dependent variables indicated in “Data analysis” section. Occasionally, gender (male, female) was used as a third factor, or independent variable, for some of the comparisons.

### Subjects and speech materials

**Figure 1:**
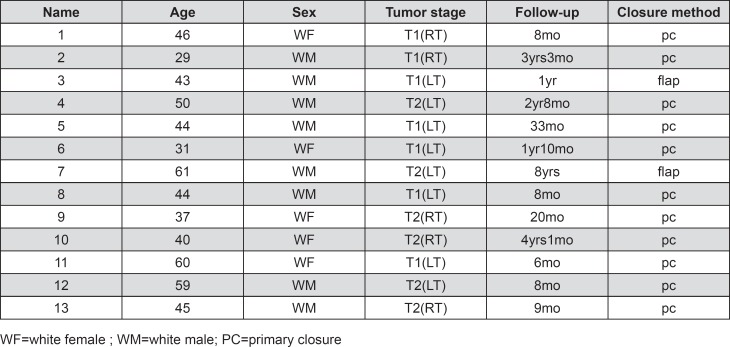
Summary of patient data

Twenty-three normal controls and 13 post-glossectomy patients (Figure 1) served as volunteers for the study. All were native speakers of American English. The control group consisted of 11 males and 12 females. The patient group consisted of eight males and five females. The average ages of the control group and patient group were 39.75 years old and 45.3 years old, respectively. All patients received a partial lateral glossectomy with no subsequent radiation or chemotherapy. Two patients underwent flap reconstruction with a radial forearm free flap (flap), the others were sutured shut with a primary closure (pc). All subjects were normal in hearing and speech perception capability. Surgeries were performed by oral and maxillofacial surgeons at the University of Maryland – School of Dentistry or by head and neck surgeons at Johns Hopkins Hospital. Subjects signed approved consent forms of the Institutional Review Board in each location.

Speech tasks were “a geese,” and “a souk.” These tasks were chosen for several reasons. They can be repeated in less than 1 second, which is within the limits of our MRI recording system. The first MRI frame is a “schwa”, which uses a fairly neutral tongue position. For “souk”, the tongue moves into the /s/ and then primarily backwards into /u/ and /k/. For “geese”, the tongue moves into the /g/ and then primarily forwards into /i/ and /s/. The words use very little jaw opening, so tongue deformation is the main component of motion and both vowels are bounded by a velar stop (/k/ or /g/) and a linguo-alveolar fricative (/s/). One patient (T1-flap) did not have acoustic data for /u/ and had no data at all for /i/, since he only recorded “a souk”. One control did not have acoustic data for either vowel due to data collection difficulties, but did have MRI data. These datasets were excluded from the related statistical analyses.

### Instruments and recording procedure

Subjects were positioned in a supine position in the MRI scanner with the neck coil positioned to image the area from the lower nasal cavity to the upper trachea.

### Audio recordings

Two audio recordings were made. The first was made prior to the MRI scan to provide good quality acoustic data for formant analysis. The subject was positioned supine in a dental chair to simulate the MRI recording position. The subject repeated each MRI word seven times and these recordings were used to measure the first three formants of the vowels /i/ and /u/. The recording was made with a head mounted short-range, unidirectional, dynamic microphone (Audiotechnica, Inc, Model AT857AMa, Tokyo, Japan) connected to an Olympus WS-500M digital voice recorder. The second recording was made inside the MRI scanner. Subjects spoke the speech tasks to a metronome before and during MRI scanning. This recording was used to segment the vowels and identify the MRI time-frames of interest. A fiberoptic subtraction microphone (Optoacoustics, Or Yehuda, Israel) captured the speech and passively subtracted the MRI noise before recording the waveform onto an Olympus WS-500M digital voice recorder. Both the metronome beats and the speech were recorded.

The metronome contained four beats. The first two were used for the two syllables of the task (a souk or a geese) and the second two were used to time an inhalation and exhalation. This controlled all motion during the MRI recording. The metronome was also used to trigger the MRI scanner so the recording began at the time of the first beat. This system was based on the one developed by Masaki, et al.^11^ (1999).

### Cine MRI recordings

**Figure 2:**
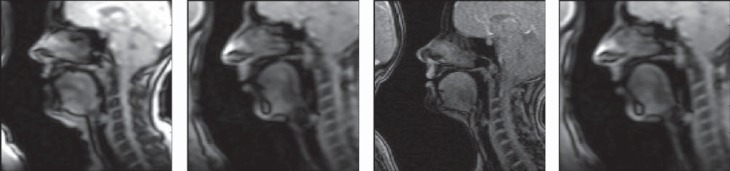
Cine MR Images for /i/ patient, /i/ control, /u/ patient, /u/ control

Cine MRI datasets were collected in multiple planes, while the subject repeated the speech tasks to the beat of the metronome. Because soft tissue produces a weak signal and the time frames are short (38 msec), multiple repetitions of the word were collected and averaged to produce a single movie. To collect a complete dataset, the subject repeated each speech task five times *per* slice. A 3-Tesla MR system (Magnetom Trio, Siemens Medical Solutions, Erlangen, Germany) was used with an eight channel head and neck coil. The parameters were: FOV=240 mm, voxel size=1.87×1.87×6.0 mm, time-frames=26. Stacks of Cine MRI images were recorded in the sagittal, coronal and axial planes (Figure 2). Depending on the size of the subject's tongue, the sagittal stack contained between five and nine slices, and the axial stack contained between 10 and 14 slices. Measurements were made from the midsagittal slice and the coronal slice that intersected the second molar, since this was encompassed by the resected region.

### Acoustic analyses

The first three formants, Fl, F2, and F3 were measured for the /i/ and /u/ in each subject using the formant tracker of Wavesurfer program. The automatically extracted formant trajectories were visually compared with spectrograms and manually corrected if any errors were detected. The linear prediction coefficients (LPC) order for formant tracking was 12 and the analysis window size was 50 ms with a shift size of 10 ms. The middle window in each vowel segment was used for the formant measurement. Each subject produced “a geese” and “a souk” seven times, and the average formant values for each subject and vowel were used in the analyses.

### Cine MRI analyses

The target vowel frame for /i/ and /u/ was defined for each subject as the midsagittal time-frame with the smallest tongue palate constriction occurring within the acoustic duration of the vowel. A coronal slice located at the second mandibular molar, mesial edge, was identified for each vowel, and the time-frame comparable with the sagittal slice was chosen for measurement. The second molar was chosen because lateral tongue cancers occur in this region and it is also the location of the high part of the palatal vault. Measurements were made from landmarks in Figure 2 using custom software written in Matlab.

**Figure 3:**
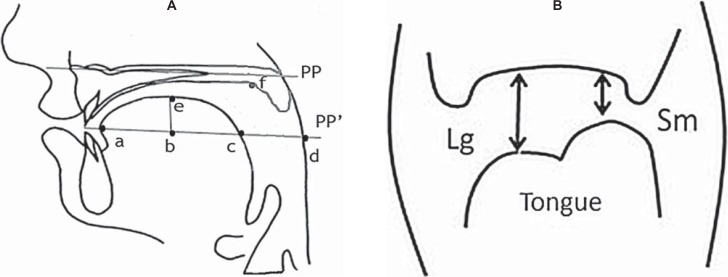
Landmarks in (A) the midsagittal plane and (B) the coronal plane at the second molar. Landmarks are based on the palatal plane (PP) and a line parallel to PP intersecting tongue tip (PP'). Tissue points used as landmarks include the tongue tip (a), the intersection of a perpendicular line drawn from point “e” to the PP' line (b), the most posterior point of tongue on the PP' line (c), the intersection point of the pharynx with the PP' line (d), the most upper point of tongue (e), palatal point closet to “f” during of /u/, /i/ pronunciation (f)

From the landmark points in Figure 3A, the following distances and lengths were measured:

AP_tng_: anterior-to-posterior tongue length on the PP' line: a – c;

AP_TOT_: distance from the tongue tip to the posterior pharyngeal wall on the PP' line: a – d;

D_pha_: distance between anterior and posterior pharyngeal walls on the PP' line: c – d;

SI_tng_: superior-to-inferior tongue height: b – e;

D_lip_: distance between upper and lower lip at minimum constriction;

D_TP_: distance between tongue and palate at the minimum constriction for /i/ and /u/. For /u/ the constriction location was more posterior than for /i/.

From the coronal landmarks (Figure 3B), the following distances were computed:

Sm: the distance between palatal mucosa and the most upper point of tongue perpendicular to the PPline, made on the side with the smaller tongue-palate distance;

Lg: the distance between palatal mucosa and the most upper point of tongue at perpendicular to the PPline, made on the side with the larger tongue-palate distance.

In some statistical analyses, ratios were used to represent important relationships. These were:

D_lip_/D_TP._: The ratio of lip constriction to tongue-palate constriction was studied to see if tradeoffs were made in constriction size, especially during the /u/, which uses two constrictions;

D_lip_/D_pha_: The ratio of lip distance to pharynx size was studied to see if tradeoffs were made between the lip and pharynx regions of the vocal tract;

SI_tng_/AP_tng_: The ratio between vertical and horizontal tongue shape was computed to determine whether patient tongue shapes indicated that different muscles were used for tongue body elevation from controls;

AP_tng_/AP_TOT_: The ratio between AP tongue length and tongue-plus-pharynx length was measured to determine whether patients had a more posterior tongue position due to the missing tissue;

Sm/Lg: Symmetry of small-to-large side tongue-palate distances was measured to corroborate that the left/right tongue size asymmetry created by the surgical resection was absent in the controls.

### Data analysis

Statistical analysis was performed using SPSS. Group, gender, and vowel were assigned as independent variables, and the dependent variables were F2/F1, F3/F2, F3/F1, D_lip_/D_TP_, AP_TOT_/AP_tng_, SI/AP_tng_, D_lip_/D_pha_, and Sm/Lg ratio. Two-way Analyses of Variance (ANOVA) were used to compare the effect of group and word, in formant values and interlip distance. Three-way ANOVAs were performed to see the effects of group, word, and gender on tongue position, tongue shape, and airway size. The level of significance was set to *p*<0.05. In addition, Pearson Product Moment Correlation coefficients were calculated between D_lip_, D_pha_, D_TP_, SI_tng_, AP_tng_, and AP_TOT_.

## RESULTS

Effect of subject group and vowel type on formant values

The patients had significantly lower F2/F1 ratios (F=5.911, p=0.018), and lower F3/F1 ratios that approached significance (F=3.482, p=0.067) (see Tables 1, 2). The ratio differences occurred because the F2 and F3 values were slightly smaller in the patients than the controls (see Table 2). This difference was seen primarily in the /u/ data. Vowel type was significantly different for all three ratios (p<.05) due to the lower F2 and F3 for /u/. The group x vowel interaction approached significance for F3/F2 (p=0.09), and was not significant for F2/F1 (p=0.849) or F3/F1 (p=0.204).

**Table 1:** Results of the formants and anatomical measurements in controls (CL) and patients (PT) for the vowels /u/ and /i/

Group-Vowel		F1 (Hz)	F2 (Hz)	F3 (Hz)	D_Lip_/D_TP_	Sm/Lg*	AP_tng_/AP_TOT_	SI/AP_tng_	D_Lip_/D_pha_
CL - u	mean	359	1746	2561	1.31	0.8	0.8	0.33	0.28
	sd	52	265	263	0.68	0.31	0.06	0.07	0.15
PT - u	mean	355	1541	2470	1.28	0.5	0.82	0.29	0.33
	sd	39	226	189	0.48	0.22	0.05	0.05	0.42
CL - i	mean	298	2497	3106	4.58	0.51	0.78	0.38	0.47
	sd	41	272	255	3.33	0.42	0.05	0.07	0.78
PT - i	mean	310	2388	2928	3.93	0.46	0.77	0.35	0.39
	Sd	39	251	268	3.13	0.3	0.03	0.06	0.62

* T1-flap did not say “a geese”, and did not have measurable acoustic data

**Table 2:** Statistical analyses and p values

Group		F2/F1	F3/F2	F3/F1	D_lip_/D_TP_	Sm/Lg	AP_tng_/AP_TOT_	SI_tng_/AP_tng_	D_lip_/D_pha_
group×word	Group	0.018	0.195	0.067	0.555	0.039	0.448	0.087	0.739
	word	0	0	0	0	0.043	0.008	0.006	0.006
	Group word	0.849	0.09	0.204	0.589	0.139	0.206	0.177	0.121
group×word×gender	group				0.701		0.499	0.087	0.892
	word				0		0.009	0.006	0.004

### Effect of subject group, word, and gender on tongue position and shape

#### Left to right tongue-palate ratios (Sm/Lg)

For patients, the side in which the glossectomy was performed had the bigger distance to the palate in the coronal plane, although some asymmetry was seen in the controls as well. Sm/Lg ratios for /u/ were 0.8±0.31 and 0.5±0.22 in controls and patients, respectively. For /i/, Sm/Lg ratios were 0.51±0.42 and 0.46±0.3 in controls and patients, respectively. The /u/ was more symmetric in controls during /u/ than /i/; patients were equally asymmetric for both vowels. These differences were statistically significant for group (Sm/Lg, F=4.426, p=0.039) and word (Sm/Lg, F=4,253, p=0.043) (Table 1).

#### Tongue shape (SI/AP_tng_)

Larger SI/AP ratios indicated a more vertical tongue shape than smaller ratios. The ratios were slightly higher for controls than patients in both vowels, and approached significance (F=3.026, p=0.087). For /u/, means and standard deviations were 0.33±0.07 in controls and 0.29±0.05 in patients. For /i/, they were 0.38±0.07 and 0.35±0.06, respectively. The ratio difference was primarily due to a lower b – e distance (SI_tng_) in the patient group. The ratio was significant for word (F=8.086, p=0.006). Gender did not show any statistical significance (F=1.863, p=0.177) (Table 2).

### Effect of subject group, word, and gender on vocal tract airway measurements

#### Pharynx size (AP_tng_/AP_TOT_, D_lip_/D_pha_)

To evaluate the Pharynx size, AP_tng_/AP_TOT_ and D_pha_ were obtained. Pharynx size showed the relative evaluation about anterior and posterior movement of tongue upon pronunciation. Upon pronunciation of /u/, AP_tng_/AP_T0T_ was 0.80±0.06 and 0.82±0.05 in controls and patients, respectively. Upon pronunciation of /i/, AP_tng_/AP_TOT_ was 0.78±0.05 and 0.77±0.03 in controls and patients, respectively. In patients, AP_tng_/AP_TOT_ was somewhat bigger, because AP_TOT_ was shown shortly and it implied that anterior and posterior movement of tongue was small. However, in statistical significance analysis, there was no statistical significance in group (AP_tng_/AP_TOT_, F=0.583, p=0.448), but there was significant in word (AP_tng_/AP_TOT_, F=7.602, p=0.008). There was no statistical significance in gender classification (AP_tng_/AP_TOT_, F=0.359, p=0.557). Upon pronunciation of /u/, D_lip_/D_pha_ was 0.28±0.15 and 0.33±0.42 in controls and patients respectively. Upon pronunciation of /i/, D_lip_/D_pha_ was 0.47±0.78 and 0.39±0.62 in controls and patients, respectively. In statistical significance analysis, there was no statistical significance in group (D_lip_/D_pha_, F=0.112, p=0.739), but there was significant in word (D_lip_/D_pha_, F=8.164, p=0.006) (Tables 1 and 2).

### D_lip_ and tongue midsagittal distances (D_lip_/D_TP_)

D_lip_ and D_TP_ were measured in the midsagittal plane and the mean was calculated. Upon pronunciation of /u/, the values of D_lip_ and D_TP_ in controls were 3.91±1.49 mm and 3.21±0.9 mm, respectively. The values of D_lip_ and D_TP_ in patients were 3.6±1.15 mm and 3.05±0.93 mm, respectively. Upon pronunciation of /i/, the values of D_lip_ and D_TP_ in controls were 7.64±2.6 mm and 2.06±0.86 mm, respectively. The values of D_lip_ and D_TP_ in patients were 6.4±2.26 mm and 2.0±0.69 mm, respectively. In general, values of D_lip_ and D_TP_ showed a slightly higher value in controls, but there was no statistically significant values (D_lip_/D_TP_, F=0.352, p=0.555) in group. However, in terms of word, there was statistically significant value (D_lip_/D_TP_, F=26.007, p=0.000), reflecting a smaller lip opening for /u/ (Tables 1 and 2).

## DISCUSSION

When the part of tongue was removed due to tongue cancer, the shape of tongue was changed and volume of tongue, which accounted for oral cavity, would be changed. The changed tongue will affect the pronunciation. Some studies reported that the damaged tissues induced the change of vocal organs after performing glossectomy, and flap reconstruction got better in order to compensate it. However, other studies reported that these flaps induced fibrosis and then interfered with tongue movement^7^. In this study, these were only two flap patients. Their speech was not noticeably worse than the primary closure patients, although physically, the long back cavity and short lip protrusion of T2-flap were among the extremes of the patients. Because flaps are often above the upper surface of the tongue, as was the case with both patients, the tongue occupies more vertical space and may lengthen the oral cavity. Both flap patients had long back cavities for /u/, only four controls and one primary closure patient had equivalent or longer back cavity lengths. However, there are not enough flap patients in this study to conclusively determine differences in the effects of closure procedure.

For studies on pronunciation of patients who underwent glossectomy, speech intelligibility, articulation, formant, and vowel space were primarily used. However, because these approaches were evaluations on pronunciation function after the surgery, there were limitations for studies on how the shape of tongue was changed after the surgery or how the tongue was changed upon the pronunciation. The present study uses Cine MRI, in which k-space data is collected over multiple repetitions of the speech utterance and an ensemble combination of the data produces a cine series of images. From midsagittal Cine MRI, one can measure the progression of tongue, lip, laryngeal, and velar motion by tracking the edges of these vocal tract structures. From these primary 2D measurements other useful quantities can be calculated, such as cavity lengths and midsagittal constriction distances. Vowel specific time frames selected from Cine MRI sequences should reveal the strategies and effectiveness of tongue motion adaptations in post-glossectomy patients, when compared with the acoustic output.

In this study, Cine MRI was used in order to investigate the changed shape of tongue and how the compensatory mechanism of tongue occurred upon pronunciation. The subjects were induced to make pronunciation and Cine MRI was recorded. The particular pronunciation was captured and the three-dimensional structure of tongue occurred upon pronunciation. We supposed that pharynx size was different between two groups in analysis of MRI, but there was almost no change in fact. Changes caused by glossectomy were Sm/Lg and SI of tongue. Changes of Sm/Lg were, of course, caused by glossectomy and SI_tng_ was shown less in the group of patients. Less SI_tng_ implied flatter tongue. Therefore, in formant analysis, F2 and F3 of group of patients showed low and the pronunciation of vowel was distorted. There was statistically significance in F2/F1 and F3/F1 values between groups (p=0.018, p=0.067). Upon pronunciation of /i/ in group of patients, the tongue tended to be flat and lip tended to be closed.

Closing the lips lower all formants, in such a way that F1 becomes normal, and F2 and F3 are low. Since F2 and F3 were shown lower in women of the group of patients, it implied that F2 upon pronunciation of /u/ and F3 upon pronunciation of /i/ were more affected in group of female patients rather than in the group of male patients. In group of male patients, F1 was increased more upon pronunciation of /i/ and it was consistent with studies of Kaji, et al.^9^ (2007). In pronunciation of /u/, D_li_, SI_tng_, and D_TP_ did not show much differences between group of patients and control group, but in pronunciation of /i/, D_lip_ and SI_tng_ were different. Since the tongue should move more upon pronunciation of /i/, a group of patients was more affected. Pronunciation and shape of tongue was changed due to glossectomy in the group of patients. Therefore, there were statistically significances in F2/F1, F3/F1, Sm/Lg, and SI_tng_/AP_tng_ between two groups.

Another important finding of this study was to quantify the relationship among tongue, lip, and pharynx upon pronunciation. In Pearson correlation analysis, D_lip_, D_TP_, SI_tng_, and D_pha_ showed statistically significant correlation (Table 3). There was correlation in D_lip_ and D_TP_ (p=0.01), SI_tng_ and D_TP_ (p=0.030), D_lip_ and SI_tng_ (p=0.000), and D_lip_ and D_pha_ (p=0.013). As D_lip_ was increased, D_TP_ was decreased. As SI_tng_ was increased, Dpha was decreased. As SI_tng_ was increased, D_TP_ was decreased. As D_TP_ was increased in group of patient, SI_tng_ had a tendency to be decreased. It implied that a group of patients had adaptation function upon pronunciation, and changes of anatomical structures affected the formant.

**Table 3:** Pearson correlation coefficient of D_lip_, D_pha_, D_TP_, SI_tng_, and AP_tng_

	D_liP_	D_pha_	D_TP_	SI_tng_	AP_tng_
D_lip_ pearson correlaton	1	.294*	.−306**	.454**	−0.076
Sig.(2-tailed)		0.013	.010*	.000*	0.531
N	71	71	71	71	71
D_pha_ pearson correlaton	294*	1	−0.059	0.022	−0.177
Sig.(2-tailed)	0.013		0.623	0.858	0.141
N	71	71	71	71	71
D_TP_ pearson correlaton	.−306**	−0.059	1	−.258*	0.07
Sig.(2-tailed)	0.01	0.623		0.03	0.563
N	71	71	71	71	71
SI_tng_ pearson correlaton	454**	0.022	−0.258	1	.242*
Sig.(2-tailed)	0	0.858	0.03		0.042
N	71	71	71	71	71
AP_tng_ pearson correlaton	−0.076	−0.177	0.07	.242*	1
Sig.(2-tailed)	0.531	0.141	0.563	0.042	
N	71	71	71	71	71
AP_TOT_ pearson correlaton	0.118	475**	0.025	0.23	.782**
Sig.(2-tailed)	0.326	0	0.837	0.054	0
N	71	71	71	71	71

* correlation is significant at the 0.05 level

** correlation is significant at the 0.01 level

The front vowel /i/ and the back vowel /u/ both require tongue body elevation, but the contact with the palate is further forward for /i/ than /u/, and therefore /i/ may be a more difficult sound for post-glossectomy patients to produce. The /i/ also requires more lateral contact between the tongue and palate and lateral glossectomy patients are missing one side of the tongue, making this task more difficult. The hypoglossal nerve enters the tongue from the rear, and divides into branches that course anteriorly. If a branch is cut, the function anterior to the cut is disabled. For /i/ a more anterior part of the tongue is elevated than for /u/. In addition, the /i/ utilizes more palatal coverage than /u/ as shown in its typical tongue-palate contact pattern. Since lateral glossectomy patients are missing tissue on one side of the tongue, adequate coverage may be more difficult for /i/. The midline date cannot reflect group differences that result from lateral features, such as degree of elevation in the lateral portions of the tongue, and lateral tongue-palate contact. It can, however, present differences in lip closure between the two vowels. The sound /i/ uses an open lip position and the sound /u/ uses protruded lips. The protruded lips cause a constriction that is an integral part of the /u/ gesture and controlled to alter the F2 frequency. The lips and tongue can trade off in such a way that more protruded lips can compensate for a less high tongue body in /u/. The results showed that lip protrusion was the only midline variable that distinguished patients from controls. Therefore, it is possible that patients have more difficulty with /i/ because they are unable to use the lips to compensate for inadequate tongue body height. This study is interested in the trade-offs between the lips and tongue during these two vowels.

Although the study was limited by the small number of patients, in particular, only two flap reconstruction patients, it provided new data that quantified the morphological changes post-glossectomy surgery, and the adaptation of the tongue and vocal tract during speech.

## CONCLUSION

Changes in lip constriction and back cavity length are likely to be compensatory, whereas midline tongue shape could be compensatory or due to post-surgical limitations. Formant changes were significant, but inaudible, suggesting that compensation was sufficient. Closure procedure appeared to have an effect on back cavity length.
